# Field enhancement of epsilon-near-zero modes in realistic ultrathin absorbing films

**DOI:** 10.1515/nanoph-2022-0816

**Published:** 2023-03-06

**Authors:** Aleksei Anopchenko, Sudip Gurung, Subhajit Bej, Ho Wai Howard Lee

**Affiliations:** Department of Physics & Astronomy, University of California, Irvine, CA 92697, USA; Photonics Laboratory, Physics Unit, Tampere University, Tampere, 33720, Finland.

**Keywords:** aluminum doped zinc oxide, epsilon near zero, field enhancement, plasmonics, zero index photonic materials

## Abstract

Using electrodynamical description of the average power absorbed by a conducting film, we present an expression for the electric-field intensity enhancement (FIE) due to epsilon-near-zero (ENZ) polariton modes. We show that FIE reaches a limit in ultrathin ENZ films inverse of second power of ENZ losses. This is illustrated in an exemplary series of aluminum-doped zinc oxide nanolayers grown by atomic layer deposition. Only in a case of unrealistic lossless ENZ films, FIE follows the inverse second power of film thickness predicted by S. Campione, et al. [*Phys. Rev. B*, vol. 91, no. 12, art. 121408, 2015]. We also predict that FIE could reach values of 100,000 in ultrathin polar semiconductor films. This work is important for establishing the limits of plasmonic field enhancement and the development of near zero refractive index photonics, nonlinear optics, thermal, and quantum optics in the ENZ regime.

## 1 Introduction

Electromagnetic field confinement and enhancement by metal nanostructures is at the core of plasmonic technologies and optical metamaterials [1–3]. Intrinsic material loss and nonlocality of the electric response of metal nanostructures limit field intensity enhancement (FIE) [3, 4] and hence present a significant challenge to real-life applications of plasmonic technologies [5, 6]. Recently, conductive materials with a vanishing real part of electric permittivity, i.e., Re(*ε*) → 0, or epsilon-near-zero (ENZ) materials, are found to be beneficial for strong field confinement in subwavelength dimensions. ENZ materials with very low (zero) intrinsic loss, i.e., Im(*ε*) → 0, have near-zero refractive index and very unusual wave dynamics [7–9]. The loss of ENZ materials has a significant impact on their optical properties. For example, loss induces the anti-Snell’s law of refraction [10, 11], but also limits performance of ENZ materials [12–14]. Several solutions have been proposed for loss reduction, e.g., use of gain-media [12] and dispersion engineering by nanostructuring [15].

Ultrathin films of ENZ materials under transverse-magnetic (TM) polarized excitation support plasmon–polariton modes [16–18], which leads to enhanced absorption and FIE. The polariton resonances in ENZ slabs with negligible-to-moderate losses, i.e., Im(*ε*) << 1, have been discussed in Ref. [19]. An analytical framework describing FIE at a local plane inside structurally symmetric subwavelength ENZ slabs has been presented in Ref. [20]. However, the analysis mainly deals with lossless and loss-compensated ENZ metamaterials and only briefly discusses the effects of losses [12, 20]. Besides, closed-form expressions for a longitudinal component of the electric field at a local plane inside the ENZ slab are given in a complex form [20]. FIE dependence on the thickness of ultrathin ENZ films has been shown by S. Campione, et al. in Ref. [18], but is limited to the lossless case. Large FIE almost independent of the film thickness is shown in a uniaxially anisotropic ENZ film with both transverse and longitudinal loss of Im(*ε*) = 0.001–0.05 [21].

Despite the importance of the FIE for ENZ optics, there is no clear physical description of the FIE dependence on the intrinsic loss and thickness of ENZ films. Therefore, in this work, using electrodynamical description of the average electromagnetic power absorbed in isotropic conducting films, we derive a clear expression for the FIE in ultra-thin ENZ films with inherent optical losses. We show the dependence of FIE on absorptance of the film due to the excitation of ENZ polariton modes, which is accessible experimentally and therefore FIE could be readily estimated. We discuss the asymptotic values of FIE in the limits of ultra-small ENZ film thickness and loss. We show that FIE is limited in the local-response approximation, i.e., with negligible nonlocal effects in the optical response [22–24]. The closed-form expressions for FIE are given for the perfect absorption due to ENZ mode excitation, the normal incidence of light and finite ENZ losses, and oblique incidence and infinitesimal losses.

## 2 Results and discussion

### 2.1 ENZ nanolayers of aluminum-doped zinc oxide

**Figure 1:**
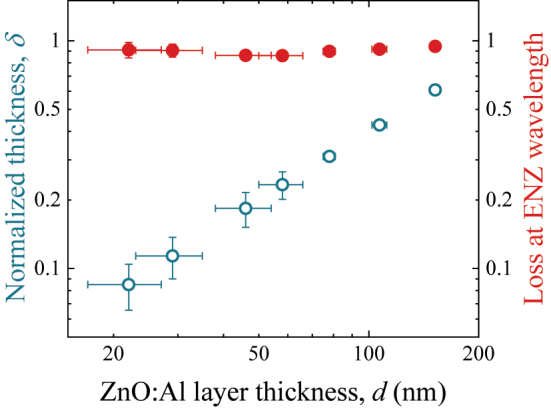
ALD ZnO:Al nanolayers. The normalized thickness (open circles, left *y*-axis) and imaginary part of the permittivity at ENZ wavelength (solid circles, right *y*-axis) obtained by ellipsometry for a series of ZnO:Al nanolayers. The thickness is normalized to the ENZ wavelength, i.e., *δ* = 2*πd*/*λ*_ENZ_. Note almost constant losses in the series; *δ* << Im(*ε*) for small ENZ layer thickness.

A series of aluminum-doped zinc oxide (ZnO:Al) nanolayers of varying thickness is grown on silica substrates using the atomic layer deposition technique (ALD). Diethylzinc (DEZ), trimethylaluminum (TMA), and deionized water are used as precursors. A temperature of 250 °C, a dopant ratio of 25:1, and a varied number of DEZ:TMA macro-cycles (which controls the final film thickness) are used in the ALD process. The optical properties and the thickness of the ZnO:Al nanolayers are obtained by spectroscopic ellipsometry in a spectral range of 400–1700 nm and angles of 55°, 60°, and 65°. The nanolayers have ENZ wavelength around 1550 nm, which increases slightly when the nanolayer thickness decreases. The thickness of the nanolayers increases linearly with the number of macro-cycles, while the imaginary part of the permittivity at ENZ wavelength has a practically constant value of 0.9 (Figure 1). A detailed description of the fabrication and characterization of ZnO:Al nanolayers is given in [25].

To confirm ENZ mode excitation, absorptance of the ZnO:Al nanolayers is measured in the Kretschmann–Raether configuration using a BK7 glass prism coupler [25]. Electric field spatial distribution profiles across the nanolayers (see Supplementary Material S1) are calculated using the transfer matrix method. The profiles were integrated over the nanolayer thickness to obtain the average FIE and compare it with the FIE obtained from the measured/calculated absorptance within the analytical framework described in the Section 2.3.1.

### 2.2 ENZ polariton modes

**Figure 2:**
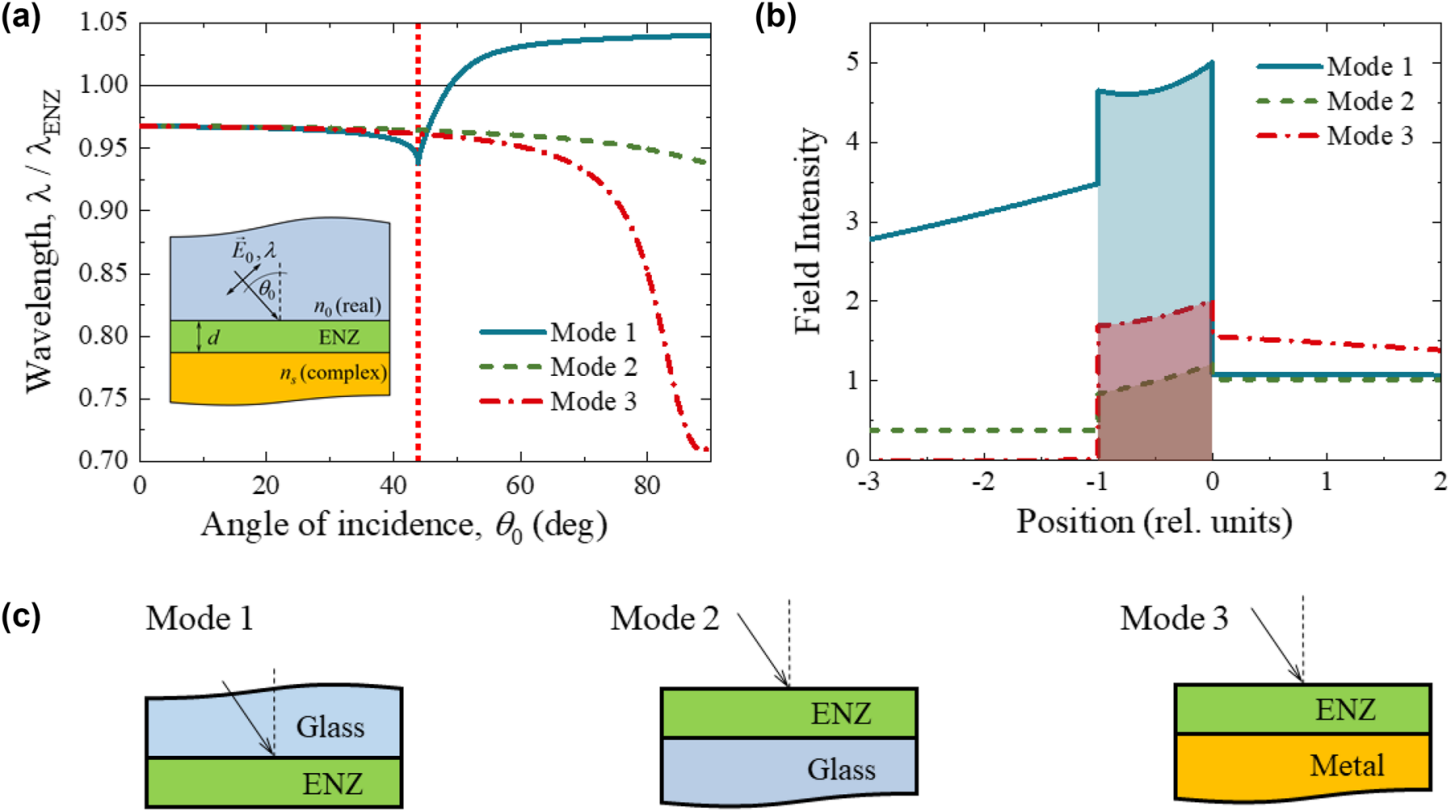
An example of ENZ polariton dispersion characteristics and FIE. (a) Dispersion characteristics and (b) electric field intensity profiles for (c) Modes 1–3 supported by a ZnO:Al nanolayer with a thickness of 58 nm. The ENZ wavelength of the nanolayer, *λ*_ENZ_, is 1562 nm. The permittivity of the ZnO:Al nanolayer is described by the Drude model. We set *n*_0_ = 1.44 (a) and *n*_0_ = 1.5 (b) for Mode 1, *n*_0_ = 1 and *n*_*s*_ = 1.44 for Mode 2, and *n*_0_ = 1 and complex *n*_*s*_ of gold for Mode 3. In part (a), the vertical dotted line shows the critical angle of 43.8° (Mode 1); inset – schematic of polariton excitation geometry and used notations. In part (b), the ENZ nanolayer reside between the positions −1 and 0; the incident wave impinging interface is at the position 0; the filled area shows the average FIE, i.e., the integral of field intensity from −1 to 0. The field profiles are shown at the ENZ wavelength and incident angle of 43.3°, 46.1°, and 52.4° for Mode 1, Mode 2, and Mode 3, respectively.

We consider an ultrathin absorbing film of permittivity *ε*(λ) = Re(*ε*) + *i* Im(*ε*) and thickness, *d*, bounded by two semi-infinite media: incident medium with refractive index *n*_0_ (real-valued) and the substrate with refractive index *n*_*s*_ (generally complex-valued) (the inset of Figure 2). The film is illuminated by TM-polarized plane wave at an angle of incidence *θ*_0_. The film thickness normalized to a wavelength of the incident light, *λ*, and the film permittivity satisfy the following conditions:

(1) $$\delta \equiv \frac{2\pi d}{\lambda }\ll 1\;\;\;and\;\;\;\left|Re\left(\varepsilon \right)\right|\ll Im\left(\varepsilon \right).$$

The last inequality along with the vanishing real part of permittivity reflects the fact that realistic ENZ media could have a high material loss. For example, ZnO:Al nanolayers have Im(*ε*) ∼ 1 at ENZ wavelength (Figure 1). The ultrathin absorbing ENZ film supports plasmon–polariton modes described by the following dispersion relationship [16, 18]:

(2) $$\left(\varepsilon_{s}\eta_{0}+\varepsilon_{0}\eta_{s}\right)\varepsilon =i\delta \left(\varepsilon^{2}\eta_{0}\eta_{s}+\varepsilon_{0}\varepsilon_{s}\xi \right),$$

where $\varepsilon_{j}=n_{j}^{2}$ , $\eta_{j}=\sqrt{\varepsilon_{j}-n_{e}^{2}}$ (*j* = 0, *s*), $\xi =\varepsilon -n_{e}^{2}$ , and $n_{e}=n_{0}\sin\left(\theta_{0}\right)$ . The solutions of Eq. (2) are sought by assuming complex-valued frequency/wavelength and real-valued wavevector/angle [26]. Depending on a value of the ratio of the refractive indexes of surrounding media, or refractive index contrast *ν* = *n*_*s*_/*n*_0_, there are three different solutions of Eq. (2):

– Mode 1 – a bound ENZ mode if both *n*_0_ and *n*_*s*_ are real and *ν* < 1 [16, 18].
– Mode 2 – a radiative ENZ mode if both *n*_0_ and *n*_*s*_ are real and *ν* > 1 [27].
– Mode 3 – a radiative Berreman mode if *n*_*s*_ is complex, e.g., the substrate is metal [17, 28].

Figure 2a shows an example of the dispersion characteristics for the Modes 1–3 given by Eq. (2) in a ZnO:Al nanolayer with a thickness of 58 nm. The permittivity of the nanolayer is described by the Drude model: $\varepsilon =\varepsilon_{\infty }-\omega_{p}^{2}/\left(\omega^{2}+i\omega \Gamma \right)$ , where *ε*_∞_ = 3.7, plasma frequency *ω*_*p*_ = 2.4 × 10^15^ Hz, and electron collision rate Γ = 2.8 × 10^14^ Hz [25]. The permittivity of gold substrate is taken from [29]. The dispersion characteristics of all three modes are close to the ENZ wavelength, *λ*_ENZ_, and vary less with the angle/wave vector when the film thickness decreases.

### 2.3 Plasmonic field intensity enhancement

#### 2.3.1 Electrodynamical description

Excitation of the ENZ modes leads to the enhanced absorptance and FIE in ultrathin ENZ films. The absorbed power per unit volume of an isotropic conducting film in harmonic electromagnetic fields with the time dependence of *e* ^−*iωt*^ can be calculated from the divergence of the Poynting vector $\vec{S}=\vec{E}\times \vec{H}$ [30, 31]:

(3) $$\begin{array}{ll} P_{\text{vol}} & =\left\langle -div\left(\vec{S}\right)\right\rangle \\ & =\frac{1}{2}\omega \left(\varepsilon_{0}Im\left(\varepsilon \right){\left|E\right|}^{2}+\mu_{0}Im\left(\mu \right){\left|H\right|}^{2}\right), \end{array}$$

where the angle brackets mean time average. Using Eq. (3), the average power absorbed by a unit area of a non-magnetic film of thickness *d* (Figure 2a) can be written as follows:

(4) $$P=\frac{1}{2}\omega \varepsilon_{0}Im\left(\varepsilon \right){\left|E\right|}^{2}d.$$

Here, |*E*|^2^ is the average (integrated over the film thickness) electric field intensity inside the film, which is different from a local field that depends on spatial coordinates. |*E*|^2^ is also the total field intensity, i.e., a sum of the filed-intensity components parallel and perpendicular to the film. The time-averaged power of a plane wave incident at an oblique angle *θ*_0_ per unit area is:

(5) $$P_{0}=\frac{1}{2}cn_{0}\varepsilon_{0}{\left|E_{0}\right|}^{2}\cos\left(\theta_{0}\right),$$

where *E*_0_ is the incident field, *n*_0_ is the refractive index of the incidence medium. From Eqs. (4) and (5), we find that absorptance is:

(6) $$A=\frac{P}{P_{0}}=\frac{\omega dIm\left(\varepsilon \right)}{cn_{0}\cos\left(\theta_{0}\right)}{\left|\frac{E}{E_{0}}\right|}^{2}=\delta \frac{Im\left(\varepsilon \right)}{n_{0}\cos\left(\theta_{0}\right)}{\left|\frac{E}{E_{0}}\right|}^{2}.$$

Therefore, the average FIE can be calculated from the absorptance using Eq. (6):

(7) $$FIE={\left|\frac{E}{E_{0}}\right|}^{2}=\frac{n_{0}\cos\left(\theta_{0}\right)}{\delta Im\left(\varepsilon \right)}A.$$

The average FIE inside the ENZ film given by the Eq. (7) is different from the field intensity at the wave-impinging boundary of the ENZ film (Figure 2b). The latter was discussed in [20]. The average FIE is the integral of electric filed intensity over the ENZ film thickness. Equation (7) establishes a relationship between FIE and absorptance of a lossy ENZ film and allows us to derive limiting behavior of FIE due to the excitation of the ENZ modes in ultrathin conducting films (see Supplementary Material S1 for the angular dependence of absorptance and FIE, and field intensity profiles for the series of ZnO:Al nanolayers).

The optical losses and thickness of the ENZ material enter Eq. (7) directly as Im(*ε*) and *δ*, but also indirectly through the absorptance *A*. In order to show the total effect of the losses and thickness on FIE we have to complement Eq. (7) with the expression for absorptance of ultrathin ENZ film. Reflection and transmission of a plane wave incident upon an ENZ medium have been studied in [10, 20, 32]. Complex reflection and transmission coefficients of an ENZ slab in the symmetric environment (*n*_0_ = *n*_s_) have been analyzed in [19, 20]. Here, we use another approach based upon the work by Abeles [33] and described in Supplementary Material S2.

#### 2.3.2 FIE of ENZ modes

**Figure 3:**
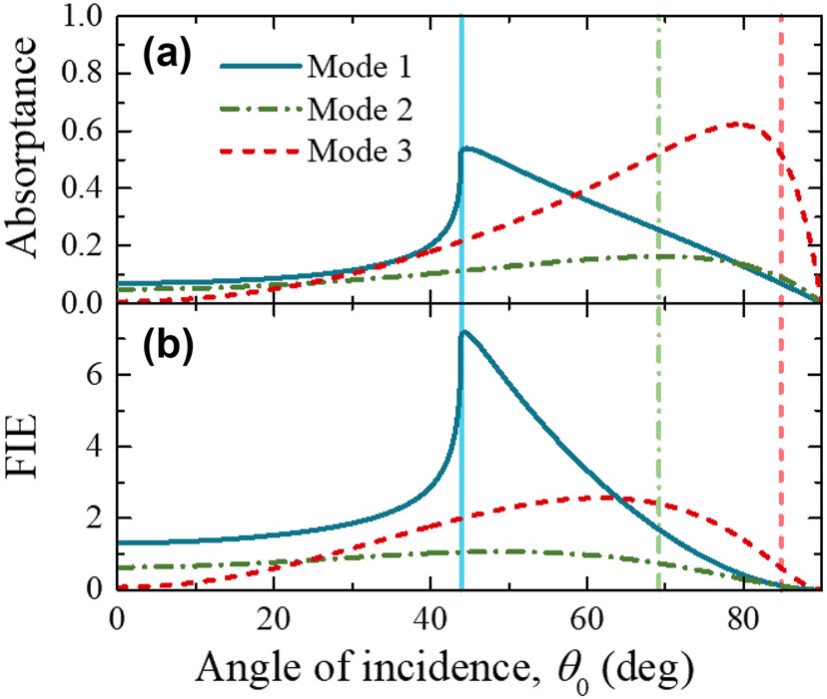
Angular dependence. (a) Absorptance and (b) FIE for a ZnO:Al nanolayer with a thickness of 22 nm at the ENZ wavelength of 1627 nm (*δ* = 0.08, Im(*ε*) = 0.9) for the three ENZ modes. The refractive indexes used in the calculations are the same as in Figure 2. The vertical lines are: the critical angle of 43.8°, the quasi-Brewster angle of 69.2°, and the pseudo-Brewster angle of 84.8°.

Using calculated or measured absorptance and Eq. (7) we can calculate FIE in ENZ films. An example of such calculations is shown in Figure 3 for a nanolayer of ZnO:Al with a thickness of 22 nm at ENZ wavelength of 1627 nm. The angular dependences of absorptance and FIE show the maxima due to the excitation of the ENZ modes. The dependence of the absorptance maxima on the thickness and loss of an ENZ film for the three modes is shown in Supplementary Material S3. For ultra-thin ENZ films, *δ* < Im(*ε*) << 1, the absorptance maximum for Mode 1 is found at the critical angle, *θ*_*c*_ = arcsin(*ν*) (Figure 3a). The angle of the absorptance maximum for Mode 2 is the quasi-Brewster angle discussed in Supplementary Material S4. For Mode 3, the angle of the absorptance maximum is the pseudo-Brewster angle for the gold-air interface. The real-valued pseudo-Brewster angle for the case of absorbing substrates (complex-valued *n*_*s*_) is given in [34]. The FIE maxima (Figure 3b) which correspond to the absorptance maxima (Figure 3a) are shifted to smaller angles because of the cos(*θ*_0_) term in the Eq. (7). In the following we analyze the dependence of the maximum attainable FIE values on film thickness and loss.

We first consider the Mode 1. In the case of lossy ENZ films, the absorptance peak approaches the critical angle when the film thickness decreases (see Supplementary Material S3). The absorptance of an ultra-thin ENZ film at the critical angle is proportional to the film thickness

(8) $$A\left(\theta_{0}\to \theta_{c}\right)=\frac{4\delta }{\delta_{th}},$$

where the film thickness satisfies the following condition:

(9) $$\delta \ll \delta_{th}=\frac{\sqrt{n_{0}^{2}-n_{s}^{2}}}{n_{0}^{2}n_{s}^{2}}Im\left(\varepsilon \right).$$

The linear relationship (9) between the threshold film thickness *δ*_*th*_ and loss is the same as the one obtained in [35, 36] for the perfect absorption in ultrathin ENZ films. Substituting Eq. (8) into Eq. (7), we find an asymptotic value of FIE achievable in ultrathin lossy ENZ films due to the Mode 1:

(10) $$FIE\left(\theta_{0}\to \theta_{c}\right)=\frac{4n_{0}^{2}n_{s}^{2}}{Im{\left(\varepsilon \right)}^{2}}.$$

**Figure 4:**
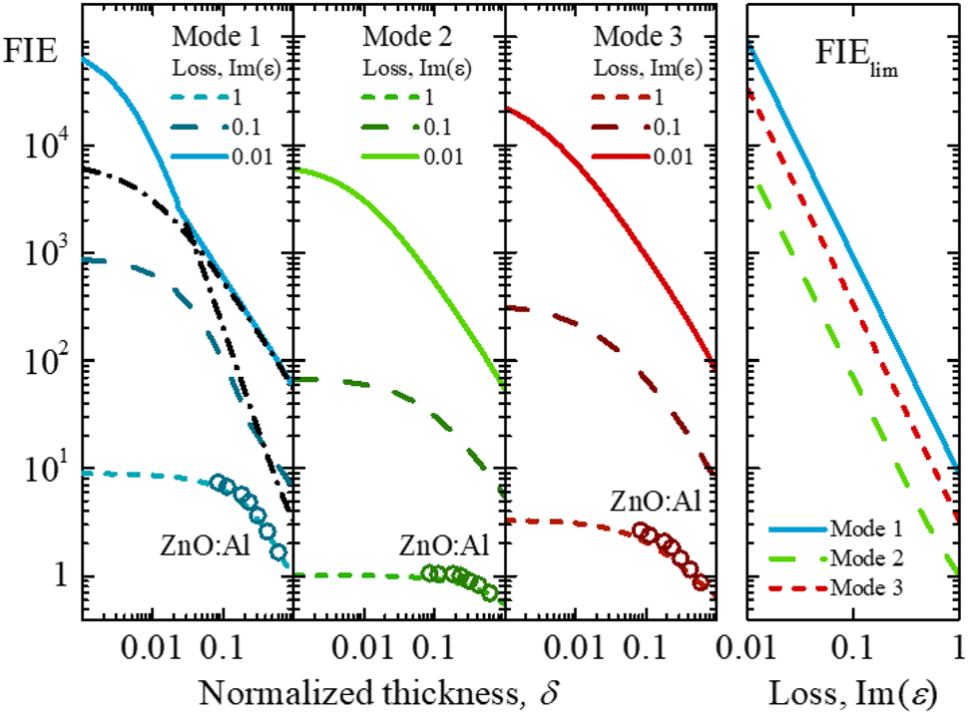
FIE in ultrathin absorbing ENZ films. (Left) Thickness and optical loss dependence of the FIE for Mode 1–3 at ENZ wavelength. The value of the imaginary part of ENZ film permittivity is shown in the legend. The refractive indexes used in the calculations are the same as in Figure 2. Open green circles show FIE data of the ZnO:Al nanolayers calculated from experimentally obtained permittivity values of ALD fabricated samples. (Right) 1/Im(*ε*)^2^ dependence of the maximum FIE approached at *δ* ≤ 0.001. The black dash-dot lines show extrapolated FIE values at the critical angle and FIE of Mode 2.

It is important that FIE is reciprocal of the second power of ENZ material losses (Figure 4). This inverse-square loss dependence could also be deduced from the continuity of the normal component of displacement field at an interface between ENZ medium and dielectric. Indeed, at the interface $\varepsilon_{0}E_{0z}=iIm\left(\varepsilon \right)E_{z}$ , so that ${\left|E_{z}/E_{0z}\right|}^{2}={\varepsilon_{0}^{2}/Im\left(\varepsilon \right)}^{2}$ , i.e., the normal component of field intensity just below the interface shows the same inverse-square dependence on ENZ loss as the average FIE of an ultrathin ENZ film (cf. Eq. (10)). In our example of ZnO:Al nanolayers, the condition (9) is satisfied for the nanolayer thicknesses smaller than 10 nm (the value of *δ*_*th*_ ≈ 0.45) and a value of the average FIE calculated from Eq. (7) for the thinnest fabricated nanolayer of 22 nm is 8 (Figure 4). This value approaches the maximum asymptotic FIE value of 11 calculated using Eq. (10) (see Supplementary Material S5). It is important that FIE could achieve values as high as 100,000 when the imaginary part of permittivity Im(*ε*) = 0.02, which follows from Eq. (10) for Mode 1 (Figure 4). This value of optical losses takes place in aluminum nitride (AlN), a polar semiconductor close to the longitudinal optical (LO) phonon frequency of 27 THz [37]. Indeed, the FIE value larger than 3600 has been shown for polaritons in ultrathin AlN films [37]. Large FIE values are predicted in optimized ZnO:Al/ZnO metamaterials with the out-of-plane imaginary part of permittivity as low as 0.003 at 1885 nm [38], and dysprosium-doped cadmium oxide (CdO:Dy) multilayers with the out-of-plane loss of 0.065 [21].

In the case of the perfect absorption, i.e., *A* = 1, the thickness of the ENZ film normalized to the perfect absorption wavelength, *δ*, satisfies the critical coupling condition [35, 36, 39, 40] and is related to the permittivity of the ENZ film and the angle of incidence as follows:

(11) $$\frac{1}{\delta }=\frac{n_{0}^{3}Im\left(\varepsilon \right)\sin\left(\theta_{0}\right)\tan\left(\theta_{0}\right)}{Re{\left(\varepsilon \right)}^{2}+Im{\left(\varepsilon \right)}^{2}}.$$

Substituting (11) into (7) gives FIE at the perfect absorption condition:

(12) $$FIE=\frac{n_{0}^{4}\sin^{2}\left(\theta_{0}\right)}{Re{\left(\varepsilon \right)}^{2}+Im{\left(\varepsilon \right)}^{2}}.$$

Therefore, at the perfect absorption conditions, FIE is inversely proportional to the second power of ENZ losses (see Eq. (1)). For Mode 1, the perfect absorption occurs at the critical angle, and FIE given by Eq. (12) is smaller than the maximum FIE given by Eq. (10) by a factor of 4. Thus, the perfect absorption is not a necessary condition for achieving the maximum FIE values (cf. Eq. (7)).

The thickness dependences of the FIE maximum for a range of loss values found in realistic ENZ media is shown in Figure 4. In the case of Mode 2 and Mode 3, the maximum absorptance is proportional to the ENZ film thickness to the first-order in *δ* (see Supplementary Material S3). Therefore, FIE is thickness independent (cf. Eq. (7)) and reaches its maximum value for the thickness *δ* << Im(*ε*). The limiting FIE values of Mode 2 and Mode 3 are smaller than the FIE value of Mode 1, Eq. (10). Also, like for Mode 1, FIE decreases with increasing losses as 1/Im(*ε*)^2^ for both Mode 2 and Mode 3.

**Figure 5:**
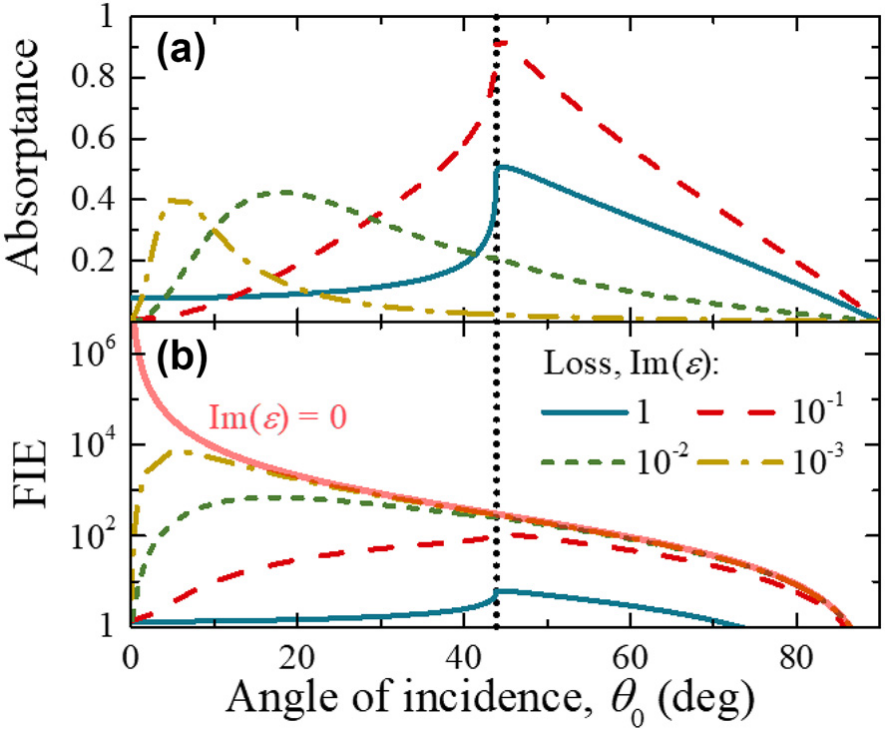
Angular dependence vs. varying ENZ loss. (a) Absorptance and (b) FIE of Mode 1 in an ultrathin ENZ film with a fixed thickness of 22 nm at the ENZ wavelength of 1627 nm (*δ* = 0.08). The varying loss is shown in the legend. In the calculations we use *n*_0_ = 1.44 and *n*_*s*_ = 1. The solid red semitransparent line in (b) shows the limiting case of infinitesimal ENZ losses of Eq. (13). The vertical dotted line is the critical angle of 43.8°.

The FIE of Mode 1, for a fixed ENZ loss, decreases as the film thickness increases and, after reaching a certain thickness threshold, decreases at a slower pace (see black dash-dot lines in Figure 4). Above the thickness threshold, the FIE thickness dependence for Mode 1 is the same as that for Mode 2, and FIE reaches its maximum value at angles smaller than the critical angle (Figure 5 and Supplementary Material S6). Figure 5 shows calculated angular dependences of absorptance and FIE in an ZnO:Al nanolayer with a fixed thickness of 22 nm and varying ENZ losses at the ENZ wavelength. As losses become smaller than the film thickness, i.e., Im(*ε*) < *δ*, the maximum absorptance and FIE occur at angles smaller than the critical angle. This agrees with the angular dependence of FIE at a local plane inside the ENZ film [20].

Now we consider the limiting case of infinitesimal ENZ losses (although realistic ENZ materials always have some loss), the absorptance become zero, and FIE approaches the following limit:

(13) $$FIE\to \frac{4{\left(\cot\left(\theta_{0}\right)\right)}^{2}}{n_{0}^{2}\delta^{2}}.$$

Equation (13) shows that, first, FIE in an ENZ film of a certain thickness becomes large and increases towards small angles of incidence as the losses become infinitesimal (Figure 5b). Second, FIE of ENZ films with infinitesimal losses scales with the film thickness as the inverse second power law at a fixed angle (Eq. (13)). The latter result agrees well with the result in [18, 20, 21]. However, in realistic absorbing ENZ films with Im(*ε*) ≠ 0, the FIE increases weaker than 1/*δ* ^2^. It has to be noted that the extent of FIE reduction due to nonlocal effects in ENZ films has yet to be quantified [23, 41], which can be done by extending our approach. Other nonlocal effects in ENZ media, such as the excitation of additional TM modes [42] or ENZ mode degeneracy lifting [43], are beyond the scope of this work.

In the case of very small but finite material losses and the normal incidence, i.e., *θ* = 0°, absorptance to the first-order approximation in *δ* is:

(14) $$A\left(\theta =0{}^{\circ}\right)\approx \delta \frac{4n_{0}Im\left(\varepsilon \right)}{{\left(n_{0}+n_{s}\right)}^{2}}.$$

From (14) it follows:

(15) $$FIE\left(\theta =0{}^{\circ}\right)=\frac{4n_{0}^{2}}{{\left(n_{0}+n_{s}\right)}^{2}}.$$

Therefore, FIE at normal incidence is small (Figure 5) and does not depend on the material losses (which, however, are finite), ENZ mode (*n*_*s*_ is real), and ENZ layer thickness if *δ* << 1.

## 3 Conclusions

We derived a clear analytical expression for the average electric-field intensity enhancement due to the radiative and bound ENZ modes in ultra-thin layers with non-negligible optical losses. We show the dependence of FIE on losses of ENZ films and the film thickness. In absorbing ENZ films, FIE reaches a finite value at the limit of deep subwavelength thicknesses. The limiting FIE value is reciprocal to the second power of the ENZ losses. Only in the case of lossless films, FIE can be described by the inverse-square law dependence on film thickness predicted by S. Campione, et al. [18]. We illustrate our analysis of the FIE dependence on ENZ film thickness with an example of ZnO:Al nanolayers of varying thicknesses grown by ALD. Our study is important for maximizing FIE in nonlinear ENZ media [44] and applications of ENZ media in quantum plasmonics [45, 46].

## Supplementary Material

Supplementary Material Details
