# Supplementary material for: Field enhancement of epsilon-near-zero modes in realistic ultrathin absorbing films
Source: Nanophotonics. 2023 Mar 6;12(14):2913–20. doi: 10.1515/nanoph-2022-0816 (PMC11501972; doi:10.1515/nanoph-2022-0816)
Supplement: Supplementary file 1 — Supplementary Material Details [file j_nanoph-2022-0816_suppl.pdf]

Supplementary Material

Aleksei Anopchenko*, Sudip Gurung, Subhajit Bej and Ho Wai Howard Lee*

Field Enhancement of Epsilon-Near-Zero Modes in Realistic Ultra-Thin Absorbing Films

Contents:

S1: Absorptance, field intensity enhancement (FIE), and field intensity profile of Mode 1 for a series of ZnO:Al nanolayers with varying thickness

S2: Absorptance calculations based upon the matrix method by Abeles

S3: The dependence of the absorptance maximum on the thickness and loss of an ENZ film

S4: The quasi-Brewster angle of the absorptance maximum for Mode 2

S5: FIE in a series of ZnO:Al nanolayers with varying thickness for the three ENZ modes

S6: The dependence of FIE on the thickness and loss of an ENZ film

***Corresponding authors:** **Aleksei Anopchenko** and **Ho Wai Howard Lee**, Department of Physics & Astronomy, University of California, Irvine, CA 92697, USA, E-mails: oleksiy.anopchenko@uci.edu, howardhw.lee@uci.edu
Sudip Gurung, Department of Physics & Astronomy, University of California, Irvine, CA 92697, USA
Subhajit Bej, Photonics Laboratory, Physics Unit, Tampere University, Tampere, 33720, Finland

Supplementary S1: Absorptance, field intensity enhancement (FIE), and field intensity profile of Mode 1 for a series of ZnO:Al nanolayers with varying thickness

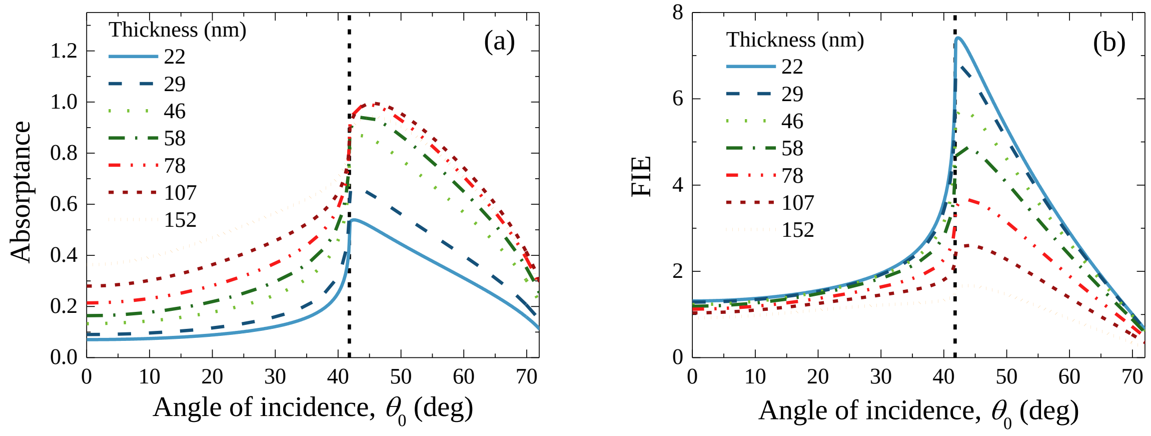

Fig. SM.1. Angular dependence of (a) absorptance and (b) FIE for a series of ZnO:Al nanolayers with varying thickness (see the legend) for the Mode 1 at the ENZ wavelength. The refractive indexes used in the calculations are: n_0 = 1.5 and n_s = 1. The vertical dashed line shows the critical angle of 41.8º. The absorptance is calculated using the transfer matrix method and optical properties and film thicknesses obtained from our ellipsometry measurements. FIE is calculated using Eq. (7) of the main text.

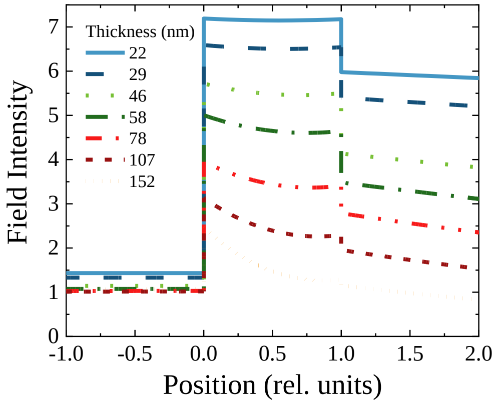

Fig. SM.2. Electric filed intensity as a function of the relative position along the film thickness for a series of ZnO:Al nanolayers with varying thickness (see the legend) for the Mode 1 at the ENZ wavelength and an angle of the maximum FIE around the critical angle of 41.8º. The wave-impinging boundary of the ENZ film has zero position. Note that the field is almost constant inside ultra-thin ENZ films and decreases as the position increases inside thick ENZ films.

Supplementary S2: Absorptance calculations based upon the matrix method by Abeles

The absorptance of ultra-thin ENZ film illuminated by TM-polarized plane wave under oblique incidence [1, 2]:

$$A = 1 - R - T\ ,\ \ R = |r|^2\text{, and } T = \left(\mathrm{Re}(Z_s)/Z_0\right)\cdot |t|^2, \tag{SM.1}$$

where R and T are the reflectance and transmittance, respectively, and $Z_j = n_j / \cos(\theta_j)$ is the wave admittance of the j-medium (j = 0, s). The complex reflection and transmission coefficients, r and t, respectively, are given by:

$$r = \frac{(m_{11} + m_{12}\cdot Z_s)Z_0 - (m_{21} + m_{22}\cdot Z_s)}{(m_{11} + m_{12}\cdot Z_s)Z_0 + (m_{21} + m_{22}\cdot Z_s)}, \tag{SM.2}$$

$$t = \frac{2Z_0}{(m_{11} + m_{12}\cdot Z_s)Z_0 + (m_{21} + m_{22}\cdot Z_s)}. \tag{SM.3}$$

Here m_{kl} (k, l = 1, 2) are the elements of the characteristic matrix:

$$[M] = \begin{bmatrix} 1 - \xi\dfrac{\delta^2}{2} & -i\dfrac{\xi}{\varepsilon}\delta \\ -i\varepsilon\delta & 1 - \xi\dfrac{\delta^2}{2} \end{bmatrix}. \tag{SM.4}$$

The reflectance and transmittance in Eq. (SM.1) are calculated at the top and bottom interfaces of ENZ film, respectively (see the inset of Fig. 2 of the main text). It is noteworthy that the dispersion characteristics of Modes 1-3 (Fig. 2 of the main text) could also be obtained by finding the poles of the complex reflection coefficient r given by Eq. (SM.2).

Supplementary S3: The dependence of the absorptance maximum on the thickness and loss of an ENZ film

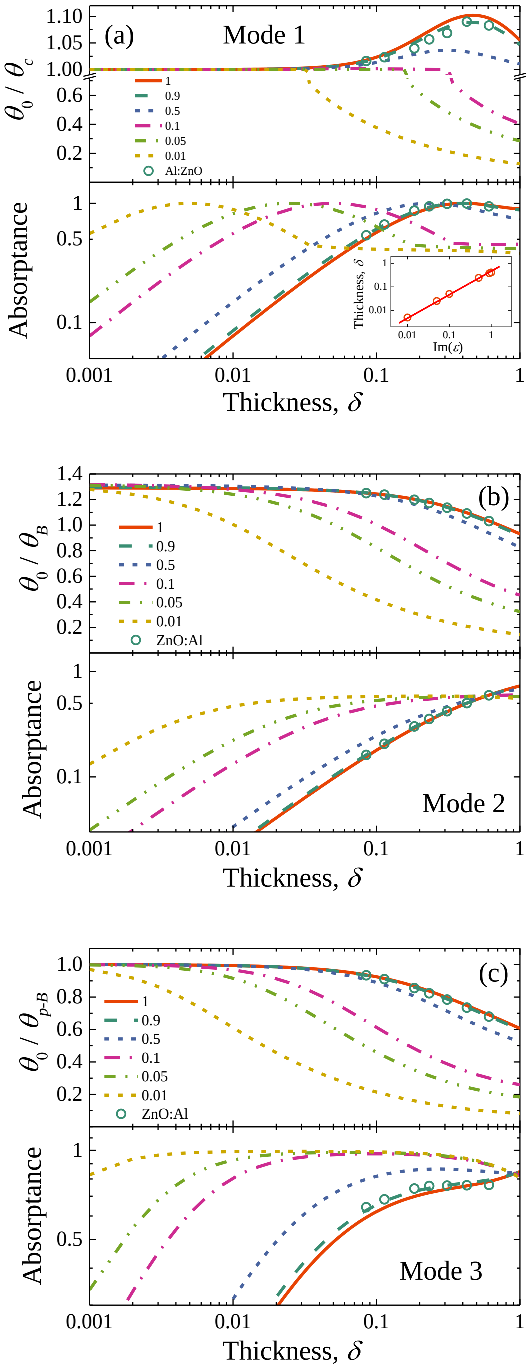

Fig. SM.3. The thickness and optical loss dependence of the absorptance maximum and its angular position for Mode 1 (a), Mode 2 (b), and Mode 3 (c) at ENZ wavelength. The imaginary part of the ENZ film permittivity is shown in the legend. The refractive indexes used in the calculations are: n_0 = 1.5 and n_s = 1 for Mode 1, n_0 = 1 and n_s = 1.44 for Mode 2, and n_0 = 1 and n_s = 0.34 + i11 (gold) for Mode 3. Open green circles show experimental absorptance data for the ZnO: Al nanolayers. The inset in (a) show a linear dependence between the loss and film thickness at the perfect absorption condition. The critical θ_c, Brewster θ_B, and pseudo-Brewster $\theta_{p\text{-}B}$ angles are used to normalize FIE angles for Mode 1, Mode 2, and Mode 3, respectively.

Supplementary S4: The quasi-Brewster angle of the absorptance maximum for Mode 2

The angle of the absorptance maximum for Mode 2 is given by the following expression:

$$\theta_{q-B} = \arccos\left[\frac{\left(\nu^2 - 1\right)\sqrt{\nu^2 + 1}}{\nu^4 + 1}\right], \quad \text{(SM.5)}$$

where ν is the refractive index contrast, $\nu = n_s / n_0$. The Eq. (SM.5) is obtained by finding a pole of the complex reflection coefficient (Eq. (9) of the main text) at a limit of zero ENZ film thickness. We will be calling $\theta_{q\text{-}B}$ as a quasi-Brewster angle. The dependence of the quasi-Brewster angle on the refractive index contrast is shown in Fig. SM.4. The quasi-Brewster angle differs from the Brewster angle, $\theta_B = \arctan(\nu)$, at low refractive index contrast, especially when $\nu \approx 1$ (Fig. SM.4). At high contrast, it asymptotically approaches the Brewster angle.

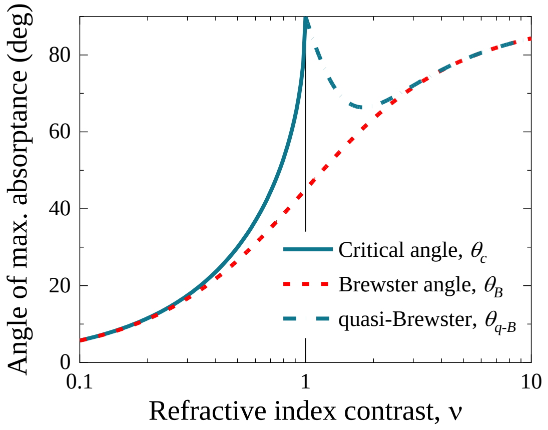

Fig. SM.4. The angle of the absorptance maximum as a function of the real-valued refractive index contrast ν. For Mode 1 ($\nu < 1$), the angle of the absorptance maximum is the critical angle, $\theta_c = \arcsin(\nu)$. For Mode 2 ($\nu > 1$), it is the quasi-Brewster angle given by the Eq. (SM.5).

Supplementary S5: FIE in a series of ZnO:Al nanolayers with varying thickness for the three ENZ modes

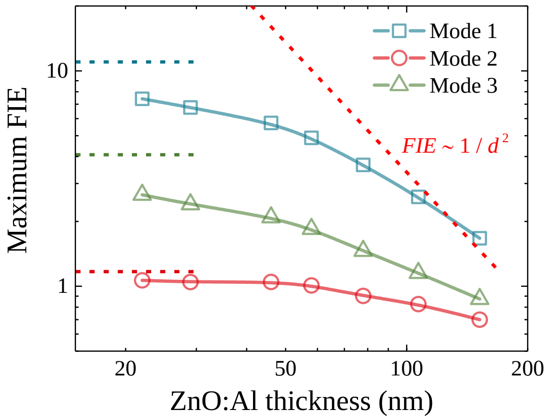

Fig. SM.5. Thickness dependence of the maximum FIE for a series of ZnO:Al nanolayers with varying thickness for the three ENZ modes at the ENZ wavelength: Mode 1 – a bound mode excited in the Kretschmann configuration (n_0 = 1.5, n_s = 1), Mode 2 – a radiative mode in the nanolayer supported by glass (n_0 = 1, n_s = 1.44), and Mode 3 – a radiative mode in the nanolayer supported by metal (gold) (n_0 = 1, n_s = 0.34 + i 11). Red dash curve shows $1/d^2$ dependence in the limit of zero losses. Horizontal dashed lines show the asymptotic values of the maximum FIE, namely 11.0, 1.2, and 4.1 for Mode 1, Mode 2, and Mode 3, respectively.

Supplementary S6: The dependence of FIE on the thickness and loss of an ENZ film

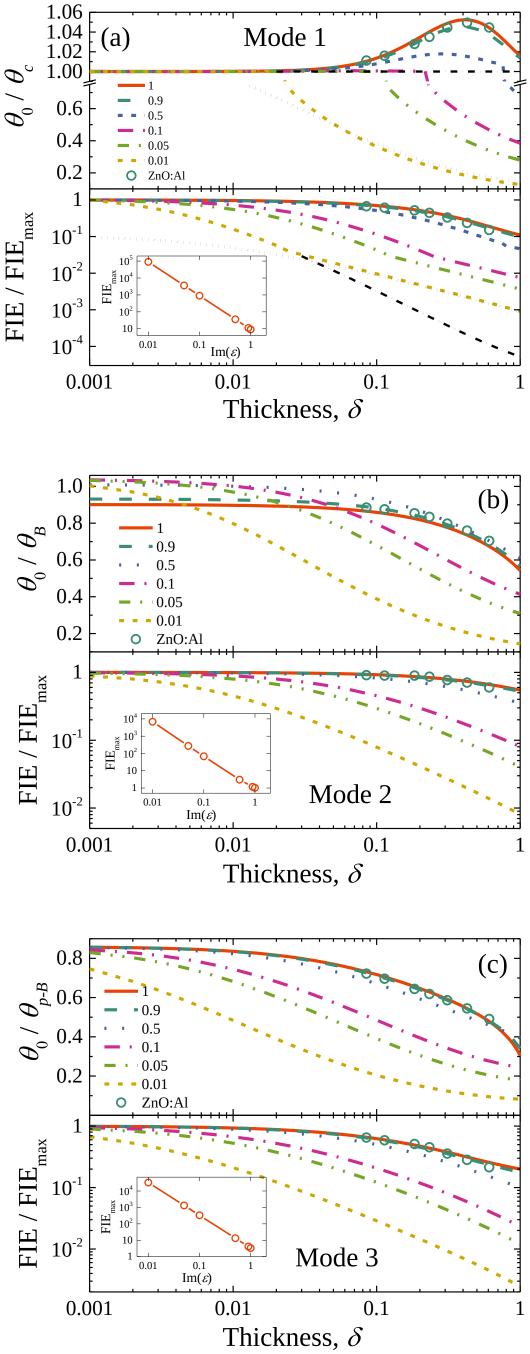

Fig. SM.6. Thickness and optical loss dependence of the FIE (angular) maximum and its angular position for Mode 1 (a), Mode 2 (b), and Mode 3 (c) at ENZ wavelength. The imaginary part of the ENZ film permittivity is shown in the legend. The refractive indexes used in the calculations are the same as in Fig. SM.3. Open green circles show FIE data of the ZnO:Al nanolayers calculated from experimentally obtained permittivity values. The insets show the $1/\mathrm{Im}(\varepsilon)^2$ dependence of the maximum FIE approached at $\delta \leq 0.001$. The maximum FIE is used to normalize FIE data. The critical, Brewster, and pseudo-Brewster angles are used to normalize FIE angles for Mode 1, Mode 2, and Mode 3, respectively. The black dash-dot line in (a) shows FIE at the critical angle, and black dot lines show FIE and angle of Mode 2 (FIE is normalized to the maximum FIE of Mode 1).

References:

[1] F. Abeles, "Optical properties of thin absorbing films," *J. Opt. Soc. Am.*, vol. 47, no. 6, pp. 473-482, 1957. doi: 10.1364/josa.47.000473

[2] W. N. Hansen, "Electric fields produced by propagation of plane coherent electromagnetic radiation in a stratified medium," *J. Opt. Soc. Am.*, vol. 58, no. 3, p. 380, 1968. doi: 10.1364/josa.58.000380
